# Supplementary material for: Conditional chemoconnectomics (cCCTomics) as a strategy for efficient and conditional targeting of chemical transmission
Source: eLife. 2024 Apr 30;12:RP91927. doi: 10.7554/eLife.91927 (PMC11060718; doi:10.7554/eLife.91927)
Supplement: Figure 3—source data 3. [file elife-91927-fig3-data3.zip › Figure 3 source data3 part 1.zip!/Figure 3-Source data 3-part1/1082-9-CRISPResso_Report_zod2ewbs.zip!/CRISPResso_on_1082_9/9.Alleles_frequency_table_around_sgRNA_catccaggcagatccaggtg.pdf]

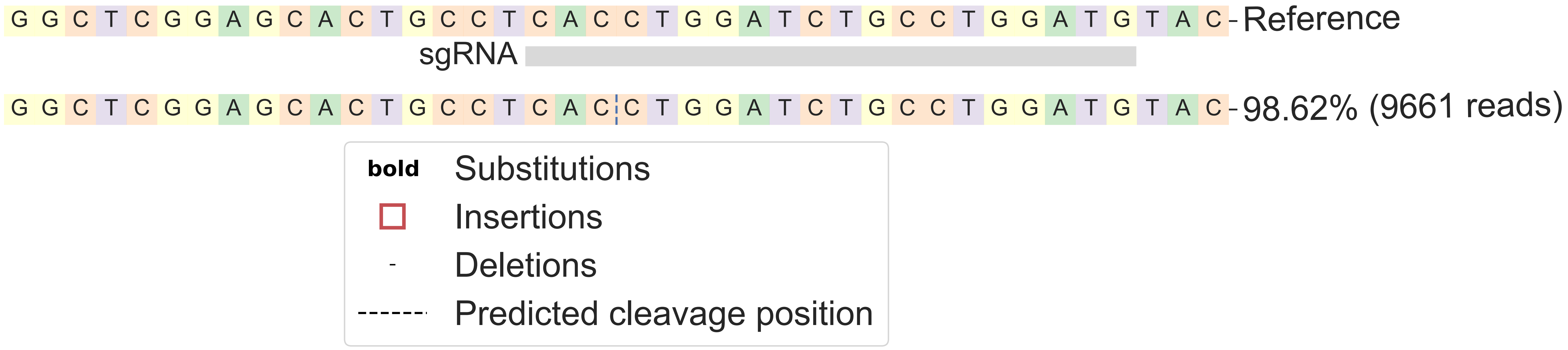

G G C T C G G A G C A C T G C C T C A C C T G G A T C T G C C T G G A T G T A C
Reference
sgRNA
G G C T C G G A G C A C T G C C T C A C C T G G A T C T G C C T G G A T G T A C
98.62% (9661 reads)
bold
Substitutions
Insertions
Deletions
Predicted cleavage position
